# Assessing Adherence to Multi-Modal Oura Ring Wearables From COVID-19 Detection Among Healthcare Workers

**DOI:** 10.7759/cureus.45362

**Published:** 2023-09-16

**Authors:** Steven K Shiba, Caroline A Temple, Joanne Krasnoff, Stephan Dilchert, Benjamin L Smarr, Janet Robishaw, Ashley E Mason

**Affiliations:** 1 Department of Internal Medicine, Florida Atlantic University Charles E. Schmidt College of Medicine, Boca Raton, USA; 2 Department of Pediatrics, Florida Atlantic University Charles E. Schmidt College of Medicine, Boca Raton, USA; 3 Department of Biomedical Science, Florida Atlantic University Charles E. Schmidt College of Medicine, Boca Raton, USA; 4 Department of Management, The City University of New York Baruch College Zicklin School of Business, New York, USA; 5 Shu Chien-Gene Lay Department of Bioengineering, University of California San Diego, San Diego, USA; 6 Halıcıoğlu Data Science Institute, University of California San Diego, San Diego, USA; 7 Osher Center for Integrative Health, University of California San Francisco, San Francisco, USA

**Keywords:** transmission, disease detection, healthcare professionals, covid-19 pandemic, oura ring, participant adherence, wearable device

## Abstract

Background

Identifying early signs of a SARS-CoV-2 infection in healthcare workers could be a critical tool in reducing disease transmission. To provide this information, both daily symptom surveys and wearable device monitoring could have utility, assuming there is a sufficiently high level of participant adherence.

Purpose

The aim of this study is to evaluate adherence to a daily symptom survey and a wearable device (Oura Ring) among healthcare professionals (attending physicians and other clinical staff) and trainees (residents and medical students) in a hospital setting during the early stages of the COVID-19 pandemic.

Methods

In this mixed-methods observational study, the data were a subset (*N*=91) of those collected as part of the larger TemPredict Study. Demographic data analyses were conducted with descriptive statistics. Participant adherence to the wearable device protocol was reported as the percentage of days that sleep was recorded, and adherence to the daily survey was reported as the percentage of days with submitted surveys. Comparisons for the primary (wearable and survey adherence of groups) and secondary (adherence patterns among subgroups) outcomes were conducted using descriptive statistics, two-tailed independent t-tests, and Welch’s ANOVA with post hoc analysis using Games-Howell.

Results

Wearable device adherence was significantly higher than the daily symptom survey adherence for most participants. Overall, participants were highly adherent to the wearable device, wearing the device an average of 87.8 ± 11.6% of study nights compared to survey submission, showing an average of 63.8 ± 27.4% of study days. In subgroup analysis, we found that healthcare professionals (HCPs) and medical students had the highest adherence to wearing the wearable device, while medical residents had lower adherence in both wearable adherence and daily symptom survey adherence.

Conclusions

These results indicated high participant adherence to wearable devices to monitor for impending infection in the course of a research study conducted as part of clinical practice. Subgroup analysis indicated HCPs and medical students maintained high adherence, but residents’ adherence was lower, which is likely multifactorial, with differences in work demands and stress contributing to the findings. These results can guide the development of adherence strategies for a wearable device to increase the quality of data collection and assist in disease detection in this and future pandemics.

## Introduction

The COVID-19 pandemic sparked interest in investigating early detection methods to combat the spread of this infectious disease, particularly among front-line healthcare workers and their patients [1]. Recent studies reported that 44% of COVID-19 infections are spread from person to person in the two-day period before symptom onset [2]. Because early detection of an impending viral infection is critical for limiting the spread of the disease, we explored the use of wearable devices that allow for real-time monitoring of physiologic indicators of health status outside a typical healthcare setting. Advances in technology have allowed companies such as Apple, Fitbit, and ŌURA to develop devices that can monitor biometrics and allow consumers to access these data through smart phones [3-21]. Changes in these metrics may be useful in detecting signs of impending SARS-CoV-2 infections [3-6,15,17,22].

However, one of the challenges of utilizing wearable devices is participant adherence, especially in the setting of health care professionals and trainees who are called upon to work long, irregular schedules and perform certain procedures incompatible with wearing a device. The Oura Ring is a wearable device that has the potential to overcome some of the challenges of adherence while collecting a variety of data. The Oura Ring collects dermal temperature, uses photoplethysmography (PPG) data to measure heart rate, heart rate variability, and respiratory rate, and estimates physical activity based on accelerometry data (recorded as metabolic equivalents; METs) as well as monitoring sleep stages. Compared to other wearables (e.g., Apple Watch, FitBit), the Oura Ring has similar or better accuracy in recording physiologic measures, along with several other advantages, including being worn on your finger, being water-resistant, having a longer battery life for seven days of continuous wear, and having a lower cost point. Additionally, at the time of the onset of COVID-19, the Oura Ring was one of the few wearable devices that measured these multiple physiologic parameters, including skin temperature [21].

Given the paucity of literature on adherence to the Oura Ring and the application of such a wearable device to a population of healthcare professionals at the time, this study aimed to investigate adherence patterns in a group of South Florida healthcare professionals and trainees during the start of the COVID-19 pandemic. Specifically, we evaluated participant adherence to a wearable device (Oura Ring Gen 2) compared to a daily symptom survey designed as part of the TemPredict Study to detect an impending infection [13,17]. Understanding the adherence patterns to various early detection tools in this specialized population will guide the development of strategies to best monitor our healthcare providers in an effort to reduce the spread of infection in ongoing and future pandemics. The ultimate aim of the larger study was to develop predictive algorithms for COVID-19. This article was previously presented as a poster presentation at the 2023 Annual Chapman Regional Conference: Caring in Action on January 14, 2023.

## Materials and methods

This observational study, which included a subset of participants within the larger University of California, San Francisco, TemPredict Study, was approved by the WCG Institutional Review Board, formerly Western Institutional Review Board (WIRB #20200974), and consisted of physiologic tracking with a daily wearable device and daily symptom survey data. The UCSF Institutional Review Board (IRB) and U.S. Department of Defense Human Research Protection Office (HRPO) approved of the TemPredict Study procedures used in the current report. The wearable device and survey portions of the study were conducted in collaboration between Florida Atlantic University (FAU) and ŌURA as part of the UCSF TemPredict Study [17], which has since demonstrated the utility of these tools in earlier detection of impending infection [17].

Study population, recruitment, and protocol

FAU-affiliated healthcare providers and trainees working at multiple clinical sites in South Florida were recruited by multiple modalities to participate in the study. Participants included healthcare professionals (FAU College of Medicine core faculty, affiliate hospital consortium faculty, and FAU student health services professionals, who are collectively referred to as healthcare professionals [HCP] in this manuscript) and trainees (resident physicians and medical students). Eligible English-speaking adult participants who owned a smartphone and were willing to co-enroll in the UCSF TemPredict Study were consented to and enrolled by the study coordinator or other trained staff. To reduce potential COVID-19 exposures, participants were sent study information electronically, followed by a virtual meeting where the protocol was thoroughly discussed, and baseline demographic information was collected. The consented participants were then met in person to retrieve an appropriately sized ring to ensure comfort and accurate physiologic readings. Training was administered on the proper use and set-up of the device, including the smart phone application, and the study protocol was reiterated. Participants were enrolled for a minimum of eight weeks.

Due to the dynamic and continuous nature of the pandemic, participants were enrolled in two phases: Phase I and Phase II. Participants in Phase I were given the option to continue their participation in Phase II. Due to safety concerns, Phase I was limited to HCPs and residents working in hospital settings, while Phase II was also open to medical students receiving training. To be recorded for daily adherence to a wearable device, participants were required to wear the Oura Ring each night while sleeping but were encouraged to wear it up to 24 hours/day. Study staff monitored participant adherence and sent frequent reminders about the protocol, which is further described below, for the duration of the study.

A total of 100 participants were enrolled in the study. Of this total, 91 participants (59% F, 41% M; mean age 36 ± 13) were included in the per protocol analysis after nine were excluded for either withdrawing their consent/participation from the study (*N*=8) or losing the provided ring (*N*=1). An additional eight participants’ symptom survey data could not be retrieved, but these participants were included in the wearable adherence portion of the study.

Data collection and storage

The physiologic data, daily symptom survey data collection, and predictive algorithm developed were addressed in detail in prior UCSF TemPredict publications [13,17]. Briefly, all participants wore the Oura Gen 2 ring device on a finger of their choosing. The Oura Ring Gen 2 collects the data and transfers it to its standard commercial mobile app (Google Play or Apple App Stores) via Bluetooth, where it is then uploaded to a cloud storage interface managed by Oura Health Oy. At the time of this study, sleep was only recorded if four hours of consecutive sleep were detected while the participant wore the ring due to the nature of the programming in the Oura Ring. Study staff had continuous access to Oura Ring data for each participant located on the cloud interface through a secure Oura Teams account. The baseline demographic data and the daily symptom survey (e.g., fever, fatigue, dry cough, unexpected loss of smell or taste, and any positive COVID-19 diagnoses provided by viral or antibody detection) adherence data were collected via a link to the UCSF Qualtrics platform that participants accessed via the Oura App on their smartphone. Survey information for each of the participants in this study was then transmitted between UCSF and FAU via a secure and encrypted data transfer process and uploaded to REDCap. Once transferred to FAU, study data were stored in the institutional Biomedical Health Research Informatics Core, which is an isolated and independently secured environment on FAU servers. Study data were managed within REDCap survey software [23,24].

Outcomes

Participants’ adherence to the wearable device protocol was defined as the percentage of nights enrolled in the study that sleep was recorded. Survey adherence was defined as a binary receipt of each daily survey and reported as the percentage of days in which a survey response was received relative to the total number of days each participant was enrolled in the study.

The primary outcome compared the above adherence patterns of the entire group of participants. The secondary outcomes compared the adherence patterns between phases and the following demographic subgroups: sex, HCP, residents, and medical students.

Statistical analysis

The physiologic data, survey data, and demographic data for each participant were analyzed in Microsoft Excel (Ver 2207, Build 16.0.15428.20182) (Redmond, USA). Participants in Phase I, Phase II, and combined populations were then organized into the aforementioned subgroups for statistical analysis. Statistical analyses of adherence were presented as comparisons by amount (N), means ± SD, or percentages. Comparisons were made between demographic subgroups, between phases, and between data types (data from a wearable device vs. symptom survey), with adherence defined as above. Adherence data were presented as percentages ± SD compared for primary and secondary outcomes utilizing two-tailed independent t-tests or Welch’s ANOVA with Games-Howell post hoc analyses and associated mean difference confidence intervals; results were deemed statistically significant at p<0.05. Standardized mean differences (Cohen’s d) and associated confidence intervals were computed for the effect size of all two-tailed independent t-tests and the Games-Howell post hoc analyses of the ANOVA.

## Results

Characteristics of study participants

The study design comprised two phases as the pandemic evolved. In Phase I, 39 participants were enrolled to complete the wearable device and survey portions of the study (36% F, 64% M; group mean age 38 ± 12). There were 71 participants in Phase II (63% F, 37% M; group mean age 37 ± 14), including 19 participants who had continued their participation from Phase I. Thus, the final study population included in the analysis consisted of 91 participants, including 35 healthcare professionals not in training, 39 residents, and 17 medical students. Table 1 shows both Phase I and Phase II cohorts had similar demographic profiles with regard to age and occupation, with the exception of medical students who were not recruited until Phase II. However, the two cohorts differed in terms of sex.

**Table 1:** The participant demographics of 91 participants were analyzed as reported on their initial enrollment. Phase I and Phase II included participants who participated in both phases. Demographics were not significantly different between Phase I and II except for sex and medical students, who were only recruited in Phase II.

	Combined* N*=91	Phase I *N*=39	Phase II* N*=71
Female *N* (%)	54 (59)	14 (36)	45 (63)
Occupation *N *(%)			
Healthcare Professionals	35 (38)	18 (46)	31 (44)
Residents	39 (43)	21 (54)	23 (32)
Medical Students	17 (19)	N/A	17 (24)
Mean Age, Years	36 ± 13	38 ± 12	37 ± 14

Adherence patterns between phases

**Figure 1:**
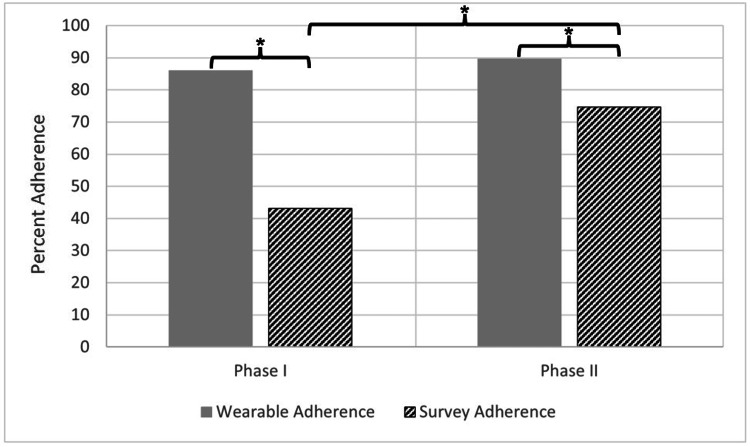
Wearable and survey adherence between phases I and II. Average percentages of nights of sleep recorded on the ring (wearable adherence) during Phase I (N=39) and Phase II (N=71) and daily surveys completed (survey adherence) during Phase I (N=35) and Phase II (N=67). * = p<0.05.

Adherence patterns were individually compared between Phase I and Phase II participants (Figure 1). For Phases I and II, wearable adherence was consistently high, with participants fulfilling the four-hour sleep requirement of 85.9 ± 12.1% and 89.7 ± 10.5% of days, respectively (t(108)=-1.63, p>0.05, d=-0.34 [95% confidence interval: -0.73; 0.06]). By comparison, survey adherence was consistently lower and differed markedly between the two phases, with participants completing daily surveys only 43.1 ± 27.1% of the time in Phase I and 74.7 ± 21.3% of the time in Phase II (t(100)=-5.99, p<0.001, d=-1.35 [-1.79; -0.90]). Thus, for both phase I and phase II, participant adherence to wearing the Oura Ring was greater than submitting daily symptom surveys (t(72)=8.61, p<0.001, d=2.08 [1.51; 2.64] and t(136)=5.21, p<0.001, d=0.90 [0.55; 1.25], respectively).

Adherence patterns overall and within demographic sub-groups

**Figure 2:**
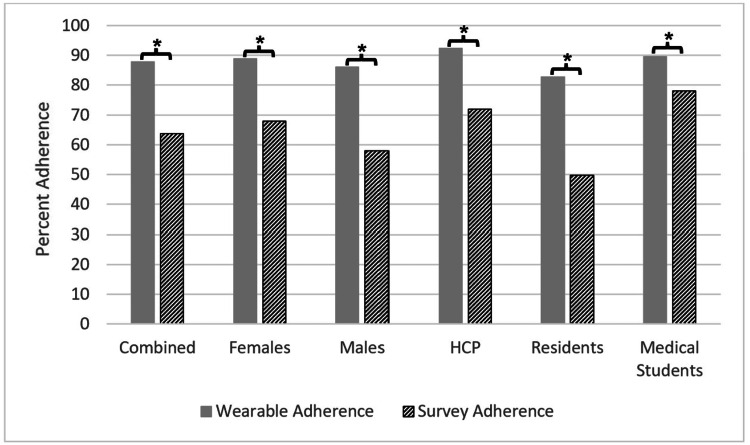
Wearable and survey adherence within each subgroup. Average percentages of nights sleep recorded on the ring (wearable adherence) stratified as all participants (*N*=91), females (*N*=54), males (*N*=37), healthcare professionals (HCP) (*N*=35), residents (*N*=39), and medical students (*N*=17), compared with daily surveys completed (survey adherence) stratified as all participants (*N*=91), females (*N*=49), males (*N*=34), healthcare professionals (HCP) (*N*=32), residents (*N*=35), and medical students (*N*=16) during combined Phase I and Phase II between subgroups. * = *p*<0.05.

Figure 2 shows participant adherence to wearable devices and survey requirements as a whole, broken down into the demographic and occupational subgroups. Within the overall group, participants demonstrated 87.8 ± 11.6% adherence to wearing the Oura Ring across study days, which was greater than the 63.8 ± 27.4% adherence to submitting daily surveys (t(172)=7.40, p<0.001, d=1.16 [0.84; 1.48]).

Within biological sex subgroups, there were also statistically significant differences when comparing wearable adherence to survey adherence. Females recorded 88.9 ± 10.8% adherence to wearing the Oura Ring compared to 67.9 ± 26.8% for daily survey adherence (t(101)=5.14, *p*<0.001, *d*=1.05 [0.64; 1.46]), whereas males demonstrated a similarly high pattern of wearable adherence (86.0 ± 12.5%) but a significantly lower daily survey adherence (57.9 ± 27.1%, t(69)=5.53, *p*<0.001, *d*=1.35 [0.84; 1.87]).

The occupational subgroups consisted of HCPs, residents, and medical students. The HCPs showed 92.4 ± 8.2% adherence to wearing the Oura Ring and 71.9 ± 23.8% adherence to submitting surveys (t(65)=4.62, p<0.001, d=1.17 [0.65; 1.69]). Medical students demonstrated a similarly high wearable adherence (89.6 ± 9.9%) as compared to survey adherence (78.1 ± 13.4%; t(31)=2.79, p<0.05, d=0.98 [0.26; 1.70]), whereas residents showed significantly lower adherence to both ring wearing (82.8 ± 12.9%) and survey submission (49.8 ± 28.7%; t(72)=6.26, p<0.001, d=1.51 [0.99; 2.03]).

In addition to adherence patterns within each subgroup, adherence patterns were also analyzed between subgroups to investigate demographic effects on adherence.

Adherence patterns between sexes

There were no significant differences between male and female adherence for each of the measures of adherence used in this study. Females recorded 88.9 ± 10.8% adherence to wearing the Oura Ring, and males recorded 86.0 ± 12.5% adherence to the wearable (t(89)=1.15, p>0.05, d=0.25 [-0.17; 0.67]). With regard to the daily survey, females were 67.9 ± 26.8% adherent and males were 57.9 ± 27.1% adherent (t(81)=1.66, p>0.05, d=0.37 [-0.07; 0.81]).

Adherence patterns between occupations

**Figure 3:**
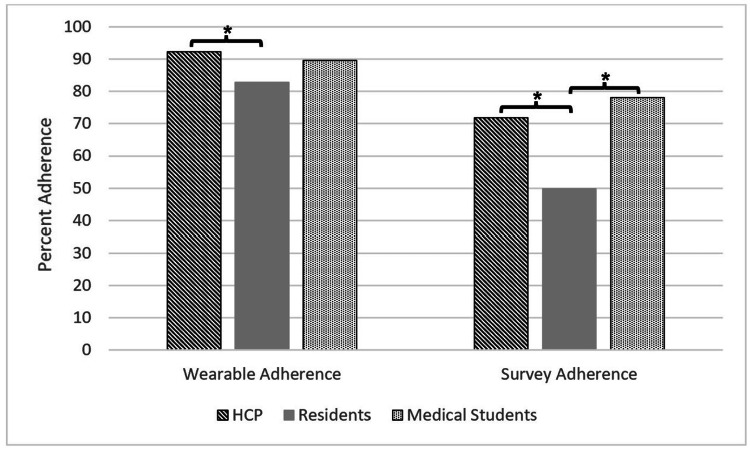
Adherence to the wearable and survey protocols between occupational groups. Comparison of healthcare professionals (HCP), residents, and medical students for each marker of adherence (sleep at night recorded on a wearable device; daily surveys completed) * = p<0.05

Figure 3 shows each occupational group compared to the others (HCPs vs. residents, residents vs. medical students, and HCPs vs. medical students) for both adherence metrics. There were differences in adherence for both the wearable and daily survey portions of the study (F(2, 42.97)=7.11, p<0.005, and F(2, 51.24)=11.12, p<0.001, respectively). HCPs were consistently more adherent than residents with regard to wearable adherence (92.4 ± 8.2% and 82.8 ± 12.9%, respectively; q(65.31)=5.37 [mean difference 95% confidence interval: 3.53; 15.62], p<0.001, d=0.88 [0.40; 1.35]), and survey adherence (71.9 ± 23.8% and 49.8 ± 28.7%, respectively; q(64.45)=4.79 [6.43; 37.75], p<0.005, d=0.83 [0.33; 1.33]). Residents did not differ from medical students in wearable adherence; they demonstrated 82.80 ± 12.88% and 89.61 ± 9.92% adherence to the wearable, respectively (q(38.64)=-2.97 [-1.10; 14.71], p>0.05, d=-0.56 [-1.14; 0.01]). However, residents were less adherent to the daily surveys when compared to medical students (49.8 ± 28.7% and 78.1 ± 13.4%, respectively; q(48.87)=-6.66 [13.77; 42.84], p<0.001, d=-1.14 [-1.77; -0.50]). There were no significant differences between HCPs and medical students for wearable adherence (92.4 ± 8.2% and 89.61 ± 9.92%, respectively; q(26.67)=1.37 [-4.30; 9.84], p>0.05, d=0.31 [-0.27; 0.90]) or survey adherence (71.9 ± 23.8% and 78.1 ± 13.4%, respectively; q(45.06)=-1.60 [-7.11; 19.55], p>0.05, d=-0.30 [-0.90; 0.31]).

## Discussion

This study assessed adherence patterns to a wearable device and daily surveys amongst a group of FAU-affiliated healthcare professionals and trainees in South Florida during the early stages of the COVID-19 pandemic. Overall, participants showed excellent adherence to both. This may reflect the high participant interest in predicting novel disease onset during an unprecedented pandemic. Consistently, however, wearable adherence (88%) exceeded that of symptom survey adherence (64%) in every comparative analysis, including all subgroups, with no statistical differences in adherence between sexes within the overall study population. Similar to the high adherence observed in a previous study [12], we attributed the high wearable adherence to multiple factors, such as emphasizing the importance of the study to participants, creating an overall environment of collaboration between researchers and participants, providing frequent feedback, and taking advantage of the ease of use of this wearable device. We further speculated that one explanation for the relatively lower symptom survey response was the requirement for the time necessary to complete it. In this regard, a recent study investigating adherence to keeping a daily diary entry reported a completion rate of 75-84% that is quite similar to that observed in the current study [25].

When comparing the sequential phases of this study, we noted a significant increase in survey adherence in Phase II relative to Phase I (74.7 ± 21.3% vs. 43.1 ± 27.1%, respectively; t(100)=5.99, p<0.001, d=1.35 [0.90; 1.79]). It is possible that during Phase II, further into the COVID-19 pandemic, participants were taking the protocol more seriously out of increased concern for their well-being. It is also possible that the difference in demographics between the two phases, namely the inclusion of medical students and an increased proportion of HCPs relative to residents, could account for the increase in adherence during Phase II.

Finally, when comparing occupational subgroups, we found the residents demonstrating consistently lower ring and survey adherence than the other two subgroups, which was even more pronounced for survey submission during Phase II. We speculate this was due in part to the increased stress and disrupted work schedules placed on residents during the pandemic, which led to overwhelming rates of hospital admissions. In this regard, medical residents were already considered to be highly stressed even before the COVID-19 pandemic, rendering them particularly vulnerable to burnout [26,27].

Altogether, this study contributes to a growing body of knowledge on the many factors influencing participant adherence, including demographics, work schedules, comfort wearing the device, and familiarity with the technology. Finally, we note that the high level of adherence of participants to a wearable device is particularly encouraging since the larger TemPredict Study showed the value of the Oura Ring in predicting the onset of COVID-19 disease among healthcare workers, which is vital in limiting disease transmission [17].

Strengths and limitations

While our study did demonstrate significant findings, there were also some limitations. The study was conducted amidst the beginning of the COVID-19 pandemic, which brought with it much unpredictability. This resulted in changes in the study demographics and protocol between the two phases. It was also a relatively small study with 91 participants. However, our cohort comprised a small subset of participants within the much larger cohort of the TemPredict Study, which had a similar demographic [8,13,17]. Although the three publications from the TemPredict Study were not focused on participant adherence, all commented on the need for participant exclusion due to missing data, which could be due to adherence issues or, at the time, issues with ensuring data capture. It should also be noted that, despite the promising aspects of wearable devices, there are also various ethical considerations surrounding their use. In addition to the principles of research ethics involved in all studies, specific concerns regarding the privacy and security of participants’ data and continuous biometric surveillance have been discussed when utilizing wearable devices [28,29]. The investigators carefully addressed these ethical concerns when planning and executing this study; researchers should take care to address these issues in wearable device studies in the future. Further, until such time that access and algorithm development are equitable across diverse populations, serious challenges to the generalizability of findings may hamper broad adoption [30]. 

There are also multiple strengths that have come from this study. Notably, the TemPredict Study led to a data-driven algorithm resulting in earlier detection of infection by an average of 2.75 days compared to conventional methods [17]. Thus, this capability, coupled with the high adherence to this wearable device shown in our study, was promising for its utility. Given the lower adherence to a daily symptom survey, wearable devices are likely a more efficacious tool in clinical practice and public health policy to reduce transmission of disease and promote the early detection of infection. Future studies and policies can be implemented utilizing this data to further expand the use of wearable devices.

## Conclusions

This study addressed the important issue of participant adherence using the Oura Ring in the setting of healthcare professionals. The results highlighted the added value of a wearable device in reaching a broader target audience with a higher rate of adherence than conventional survey tools. Such findings can help guide protocol development for future studies utilizing wearable devices as part of their protocol to increase the quality of data collection. The high adherence to the wearable device was promising for its use in public health policies and other clinical applications for the early detection of disease.
